# Technological perspectives on laser speckle micro-rheology for cancer mechanobiology research

**DOI:** 10.1117/1.JBO.26.9.090601

**Published:** 2021-09-21

**Authors:** Zeinab Hajjarian, Seemantini K. Nadkarni

**Affiliations:** Harvard Medical School, Massachusetts General Hospital, Wellman Center for Photomedicine, Boston, Massachusetts, United States

**Keywords:** laser speckle micro-rheology, micro-mechanical imaging, cancer biomechanics, tumor mechanobiology

## Abstract

**Significance:** The ability to measure the micro-mechanical properties of biological tissues and biomaterials is crucial for numerous fields of cancer research, including tumor mechanobiology, tumor-targeting drug delivery, and therapeutic development.

**Aim:** Our goal is to provide a renewed perspective on the mainstream techniques used for micro-mechanical evaluation of biological tissues and biomimetic scaffoldings. We specifically focus on portraying the outlook of laser speckle micro-rheology (LSM), a technology that quantifies the mechanical properties of biomaterials and tissues in a rapid, non-contact manner.

**Approach:** First, we briefly explain the motivation and significance of evaluating the tissue micro-mechanics in various fields of basic and translational cancer research and introduce the key concepts and quantitative metrics used to explain the mechanical properties of tissue. This is followed by reviewing the general active and passive themes of measuring micro-mechanics. Next, we focus on LSM and elaborate on the theoretical grounds and working principles of this technique. Then, the perspective for measuring the micro-mechanical properties via LSM is outlined. Finally, we draw an overview picture of LSM in cancer mechanobiology research.

**Results:** With the continued emergence of new approaches for measuring the mechanical attributes of biological tissues, the field of micro-mechanical imaging is at its boom. As one of these competent innovations, LSM presents a tremendous potential for both technical maturation and prospective applications in cancer biomechanics and mechanobiology research.

**Conclusion:** By elaborating the current viewpoint of LSM, we expect to accelerate the expansion of this approach to new territories in both technological domains and applied fields. This renewed perspective on LSM may also serve as a road map for other micro-mechanical measurement concepts to be applied for answering mechanobiological questions.

## 1 Introduction

It is increasingly recognized that the micro-scale viscoelastic properties of tissue confer critical micro-mechanical cues that orchestrate nearly all aspects of cellular function, including growth and differentiation.^1^^,^^2^ This tightly controlled, viscoelastic micro-environment is essential to organ development, wound healing, and normal homeostasis in biological tissues.^3^^–^^5^ Irregular micro-mechanical remodeling of tissue is implicated in a broad spectrum of pathologies, including cardiovascular disease, fibro-proliferative disorders, hematological diseases, and cancer.^1^^,^^6^^,^^7^ Moreover, meticulously tuned deformability and compliance are imperative for the design of synthetic scaffolds and hydrogels, to mimic normal and pathological structure and function of the tissue microenvironment, and to adjust the release of nutrients, oxygen, reagents, and therapeutics in drug-delivery applications.^8^

The development of novel tools for measuring the viscoelastic properties of tissue at microscopic length scales is instrumental for understanding the mechanical attributes of cellular structures and their microenvironment and identifying their relationships to the progression of tumor malignancies. These tools are also imperative for staging of the diseases, based on significant mechanical contrast between normal and abnormal tissue states, guiding drug delivery, and devising new lines of therapeutic strategies that target micro-mechanical aberrations.

The complex micro-mechanical milieu of tissues may be characterized by the shear viscoelastic modulus, $G^{*}(\omega )=G^{\prime }(\omega )+jG^{\prime \prime }(\omega )$, where $G^{\prime }$ and $G^{\prime \prime }$ are the elastic and viscous moduli, respectively, and ω is the angular loading frequency.^9^ Mechanical rheometry is the traditional approach for evaluating the $G^{*}(\omega )$ of materials. In this invasive process, the specimen is sandwiched between two parallel plates, and the sinusoidal shear strain, ε(ω), is applied to the specimen. This induces an oscillatory stress to the sample, σ(ω). The rheometer evaluates the twisting and displacements of the plates and calculates the $G^{*}(\omega )$, as the stress-to-strain ratio, i.e., σ(ω)/ε(ω), for a limited frequency range. Averaging the cumulative response over the whole sample volume in the rheometer obscures the mechanical heterogeneities, and this traditional tool returns only the bulk mechanical response of the specimen. Moreover, this approach calls for a relatively large sample volumes and is not conducive for rare and precious biomaterials and tissue specimens.^10^

The need for non-invasive micro-mechanical testing has motivated the development of several innovative technologies for evaluating the micro-scale viscoelastic properties of tissues and biomaterials. These techniques involve a variety of “active” approaches that measure tissue response to an extrinsic force, and “passive” approaches that rely on thermal fluctuations within tissues to probe micro-mechanical behavior. Here, we briefly review a variety of active mechanical testing approaches and focus our attention on evolution of passive techniques as novel means for non-invasive micro-mechanical assessment. In particular, we detail our perspectives on laser speckle micro-rheology (LSM), a paradigm for realizing micro-mechanical characterization and elaborate on new basic and translational opportunities in cancer mechanobiology research.

## 2 Measuring Micro-Mechanics

### 2.1 Active Methods

Active micro-mechanical sensing methods frequently involve physical contact with the specimen to apply external forces to induce displacement and deformation. These active methods frequently involve sophisticated instrumentations, with the advantage of applying large forces to probe stiff materials beyond the linear viscoelastic regime.

The conventional tool for measuring the micro-scale viscoelastic properties of materials is the atomic force microscopy (AFM)-based indentation. In AFM, a flexible cantilever harboring a micron-sized tip is mounted on a precision stage that tracks the tip displacement as it pokes the specimen. Optical position sensing is used to retrieve the cantilever deflection and calculate the force. Fitting an appropriate model to the force–distance curve yields the elastic indentation modulus (E) of the specimen.^11^ Due to its contact-based nature, AFM is inherently invasive. Because the measurements are exuberantly long, they are frequently limited to infinitesimal field of views of a few 10s of $\mu m^{2}$. Moreover, AFM only reflects the mechanical properties over depths of 10 s of μm. In addition, AFM measurements are subject to adhesion and other electrostatic forces. Finally, AFM-based indentation yields only the elastic modulus at a fixed indentation rate and does not probe the frequency dependence or the dynamic viscosity. To circumvent these limitations, several innovative tools are developed for evaluating the micro-mechanical properties of cells and their microenvironment.

For example, magnetic twisting cytometry (MTC) evaluates the force transduction of cell membrane to measure the viscoelastic remodeling of cells.^12^^,^^13^ In MTC, an oscillatory magnetic torque is applied on a magnetic bead, bound to a transmembrane receptor. Bead rotation induces an oscillatory twisting stress within the cell. A magnetometer evaluates the $G^{*}(\omega )$ of the cell as the ratio of rotary torque to bead rotation.^12^ MTC was initially used for demonstrating the integrin-mediated cellular mechano-transduction.^12^ Later, MTC measurements of bronchial epithelial cells yield the $G^{*}(\omega )$ in $\omega =0.03\text{–}16\;s^{-1}$ range ($s^{-1}=\mathrm{Hz}$) and revealed a weak power-law behavior of $G^{*}∼\omega^{\alpha }$, α=0.25.^13^ The loss tangent ($\tan\delta =G^{\prime \prime }/G^{\prime }$) was 0.5 and varied minimally with frequency, indicating a predominantly elastic behavior.^13^ The frequency dependence and loss tangent may be readily evaluated in MTC. However, since the bead–cell contact area is not precisely known, this technique is mostly qualitative. In addition, frequency range is limited by sampling rate of magnetometers.^13^

Optical tweezer active micro-rheology (OTAM) is another active technique that employs a highly focused laser beam to trap small dielectric particles.^14^ By moving the beam, particles apply a local stress to the surrounding material. Sinusoidal oscillation of the beads in the optical trap and tracing the nano-meter scale motion of the bead yield local $G^{*}(\omega )$ over a wide range of ω=3 to $15,000\;s^{-1}$ ($s^{-1}=\mathrm{Hz}$).^14^ OTAM has been applied for investigating the cell-extra-cellular matrix (ECM) micro-mechanical coupling and hemostasis and showed that at high frequencies ($\omega >400\;s^{-1}$), and $G^{*}$ exhibits distinct power-law behaviors, i.e., $G^{*}∼\omega^{\alpha }$, within cells (α∼0.7) and the ECM (α∼0.5).^14^ Moreover, the frequency, $\omega_{c}$, at which the mechanical properties transition from elastic to viscous (i.e., $G^{\prime \prime }/G^{\prime }=1$) is drastically smaller in cells (2 kHz) compared to ECM (7 kHz).^14^ Despite its wide frequency range, the small magnitude of applied forces (pN range) limits this approach to compliant specimens. In addition, because of the high NA, local heating and phototoxic effects are inevitable.

Quantitative micro-elastography (QME) is a novel variant of compression optical coherence elastography (OCE)^15^ The parent compression OCE that maps the tissue strain/deformation is response to a static applied force and provides a qualitative elastogram.^16^ By incorporating a compliant stress sensor, QME permits mapping the absolute Young’s modulus. QME exhibits up to 100-fold contrast improvement by distinguishing the tissue components that have similar deformation but distinct elasticities.^15^ In highly heterogeneous tissue, the stress varies with depth, violating the QME assumptions.^17^ Inverse methods may be used to back out the stress distribution in the tissue volume, based on the surface stress, depth-resolved strain, and micro-structural information afforded by the baseline OCT images.^18^ Because the gradient of displacement is calculated over multiple pixels along the depth, axial resolution of QME is reduced 5 to 10 times compared to the underlying OCT to ∼100  μm.^19^ The lateral resolution is also reduced because the incompressible stress sensor translates a step change in elasticity within the sample (i.e., a sharp feature boundary) to a stress gradient. As such, the stress–strain ratio presents a blurred map of elastic modulus, particularly in deeper sections.^16^

### 2.2 Passive Methods

Endogenous motions and vibrations may also be used as a source of deformation, enabling all-optical, non-contact mapping of the viscoelastic properties within the biological tissue.^10^ For passive measurements, materials must be sufficiently soft to permit detectable motions in response to subtle internal fluctuations. The passive methods may be advantageous compared to active techniques in that the measurements are within the linear viscoelastic regime.^20^ In addition, because passive methods do not require a loading mechanism, the instrumentation is often simplified, and the hardware frequently has a smaller footprint compared to active techniques. Moreover, since no physical contact is needed, these methods are often non-invasive. Here, we review some of the prominent passive techniques.

Brillouin microscopy is based on the inelastic scattering of light, due to its interaction with the local spontaneous acoustic waves within the tissue.^21^ Brillouin frequency shift is proportionate to the velocity of these intrinsic acoustic vibrations, which are in turn proportionate to the square root of the Young’s modulus at the GHz frequency range, with the exact relationship being specific to the tissue or cell type.^22^ Implemented using a confocal geometry, Brillouin microscopy provides a non-destructive, label- and contact-free method for probing the viscoelastic properties of biological samples with diffraction-limited axial and lateral resolutions (1.5 and 0.3  μm), as demonstrated in the context of *in-vivo* tissue and *in-vitro* cell levels.^22^^,^^23^

Particle tracking micro-rheology (PTM) is another passive approach that involves injecting fluorescently labeled sub-micron beads into the cytoplasm of live cells.^24^^,^^25^ High-frame rate fluorescent video microscopy is used to track the motion of particles. Statistical averaging of particle trajectories yields the mean-square displacement (MSD) $\left\langle \Delta r^{2}(t)\right\rangle$.^24^ Replacing the MSD in the generalized Stokes–Einstein relation (GSER) returns the complex frequency-dependent $G^{*}$ of the cytoplasm over the frequency range of 0.1 to $10\;s^{-1}$.^26^ The upper frequency is limited to the rate at which the particle position is recorded, i.e., the frame rate of the camera.^25^ Because PTM permits obtaining the entire particle trajectory, it is further possible to analyze the individual particles’ trajectories beyond the spatially averaged MSD and gain insight into the local micro-rheology of the cytoplasm.^10^^,^^26^

Dynamic light scattering (DLS) may also be used for evaluating the Brownian displacements of extrinsic particles.^27^ In DLS, time-dependent intensity fluctuations of light, that is singly scattered from extrinsic particles, are collected through a pinhole, captured by a photodiode, and analyzed by a digital correlator.^20^^,^^27^ Due to the single-scattered nature of collected light, the light intensity fluctuates very slowly and needs to be analyzed for long times ($10^{4}$ or tens of thousands of seconds), to detect perceptible MSD of the tracer particles.^10^ In addition, the sample must be transparent, homogeneous, and ergodic, so that the extensive temporal averaging provides a statistically accurate ensemble averaging.^10^ Moreover, large particles’ displacements are needed to sufficiently change the optical path length and provoke a noticeable phase shift and a detectable speckle intensity variation, yielding DLS insensitive to smaller MSD of particles in stiffer samples.^27^ Development of the diffusing wave spectroscopy (DWS) enabled evaluating the opaque, highly multiple-scattering media, where light propagation is nearly diffusive.^27^ Nevertheless, DLS and DWS are limited to the extremes of transparent or highly opaque specimens.

LSM is a novel optical approach that affords mapping the micro-scale viscoelastic properties of biomaterials and tissues without using extrinsic particles, in a passive, non-invasive manner.^28^^–^^30^ Speckle is a grainy intensity pattern that forms by the self-interference of coherent laser beam, as it backscatters from the turbid materials, including opaque tissue.^31^ Thermal Brownian motion of intrinsic light scattering particles continuously alter the relative optical phase shifts of backscattered rays and provoke temporal speckle intensity fluctuation. These fluctuations are exquisitely sensitive to particle displacements, and in-turn to the viscoelastic properties of the tissue particles’ microenvironment.^6^^,^^28^^,^^30^^,^^32^^–^^38^ Because in LSM light is multiply scattered from the tissue particles, even minute motions (fraction of a wavelength, in the order of nm) of particles, encountered within each lightpath, give rise to cumulative phase shifts that induce perceptible speckle intensity fluctuations. The acute susceptibility of speckle intensity to particle displacements as small as a few angstroms in LSM makes this technique capable of characterizing the mechanical properties in stiffer samples, in which particle displacements are reduced to subtle vibrations.

## 3 Laser Speckle Micro-Rheology: Technological Perspectives

### 3.1 Principles of Operation

**Fig. 1:**
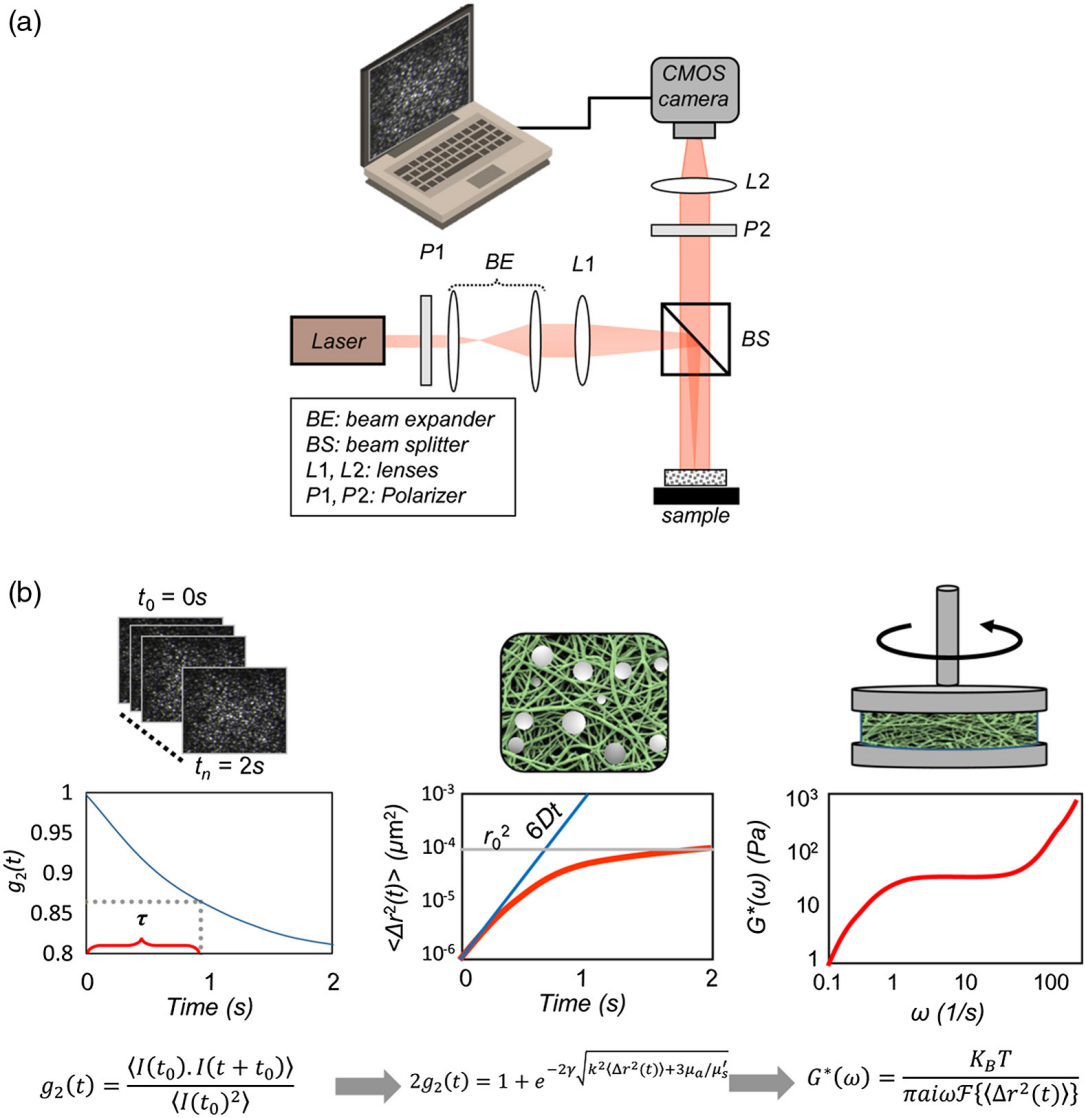
(a) Schematic diagram of a typical (LSM) setup. A laser beam is directed to the sample surface via a linear polarizer (P1), beam-splitter (BS), and a focusing optics (BE, $L_{1}$). The cross and co-polarized speckle patterns are imaged by a macro-lens (L2), on to a high-speed CMOS camera sensor. Captured speckle time series are transferred to a computer for processing. (b) The LSM concepts used to measure the shear viscoelastic modulus, $G^{*}(\omega )$ from the speckle intensity fluctuations. Cross-correlation of speckle frame series provides the speckle intensity auto-correlation function, $g_{2}(t)$, from which MSD is deduced. Substituting the MSD in GSER yields the $G^{*}(\omega )$. (This figure is reproduced with modification from Hajjarian and Nadkarni.^30^)

A typical LSM optical setup is displayed in Fig. 1(a).^6^^,^^28^^,^^32^^–^^35^^,^^38^^,^^39^ In short, a polarized laser beam is focused on the sample surface. The backscattered speckle patterns are filtered by a linear polarizer and focused by a macro-lens on to a high-speed CMOS camera sensor.^6^^,^^28^^,^^32^^–^^35^^,^^38^^,^^39^ The choice of camera frame rate and acquisition time are motivated by the range of frequencies of interests, over which the $G^{*}(\omega )$ is to be evaluated. They are further adjusted according to the sample dynamics and relaxation times, such that the speckle patterns exhibit a high contrast between dark and bright spots, and that the intensity autocorrelation curve fully decorrelates well before the end of acquisition.^6^^,^^32^^–^^34^

In LSM, quantitative analysis of the speckle dynamics returns the viscoelastic properties of the tissue microenvironment. The working principles of LSM are displayed in the flowchart of Fig. 1(b) as previously detailed in several publications. Briefly, the speckle intensity temporal autocorrelation curve, $g_{2}(t)$, is obtained by measuring the correlation between pixel intensities over the time series of speckle images according to the following equation:

$$g_{2}(t)=\frac{\left\langle I(t_{0}).I(t+t_{0})\right\rangle }{\left\langle I{\left(t_{0}\right)}^{2}\right\rangle },$$ (1)

where $I(t_{0})$ and $I(t+t_{0})$ are the intensities of individual pixels at times $t_{0}$ and $t+t_{0}$, respectively, and ⟨ ⟩ represents spatio-temporal averaging over the ensemble of all pixels in the frames and over all reference frame times, $t_{0}$. From the $g_{2}(t)$ curve, the MSD, i.e., $\left\langle \Delta r^{2}(t)\right\rangle$, of scattering particles may be retrieved. For low viscosity liquids, scattering particles simply diffuse throughout the sample and this random motion corresponds to a linear trend for MSD. On the other hand, in highly elastic solids, the particle displacements reduce to sluggish vibrations around the equilibrium, which is evidenced by a saturated MSD.^32^^,^^34^^,^^35^ For viscoelastic materials, the MSD curve takes on more complex forms as a function of the lag time. Once the MSD is evaluated, the GSER is used to extract the complex shear modulus, as a function of frequency as follows:

$$G^{*}(\omega )=\frac{K_{b}T}{a\pi i\omega F\langle (\Delta r^{2}(t))\rangle },$$ (2)

where F is the Fourier transform and a is the scattering particle radius. Moreover, $K_{B}$ stands for the Boltzmann constant ($1.38\times 10^{-23}\;m^{2}\;\mathrm{kg}\;s^{-2}\;K^{-1}$), T is the temperature (degrees Kelvin), and ω corresponds to the frequency at which the $G^{*}$ is evaluated, and conceptually parallels the mechanical oscillation frequency in the conventional rheometry as detailed above.^6^^,^^20^^,^^33^^–^^35^ Due to the discrete and finite nature of the data points, and to avoid numerical errors at frequency limits, an algebraic approximation of Fourier transform is frequently used to calculate the $F(\langle \Delta r^{2}(t)\rangle )$. This is achieved by fitting the MSD to a power-law form, i.e., $\langle \Delta r^{2}(t)\rangle \propto t^{\alpha (t)}$, where $\alpha (t)=|\frac{\partial \;\ln\langle \Delta r^{2}(t)\rangle }{\partial \;\ln\;t}|$ is the log–log slope of MSD. Subsequently, the complex $G^{*}(\omega )$ may be expressed as

$$G^{*}(\omega )={\frac{K_{b}T}{\pi a\Gamma (1+\alpha (\omega ))\Delta r^{2}(\omega )}|}_{\omega =\frac{1}{t}}(\cos(\frac{\pi \alpha (\omega )}{2})+i\;\sin(\frac{\pi \alpha (\omega )}{2})).$$ (3)

The equation above returns both the elastic (storage) and viscous (loss) moduli, i.e., the real and imaginary parts of the modulus. Here, Γ is the gamma function.^6^^,^^20^^,^^33^^–^^35^

### 4.4 Influence of Optical Properties and Scattering Particle Size Distribution

**Fig. 2:**
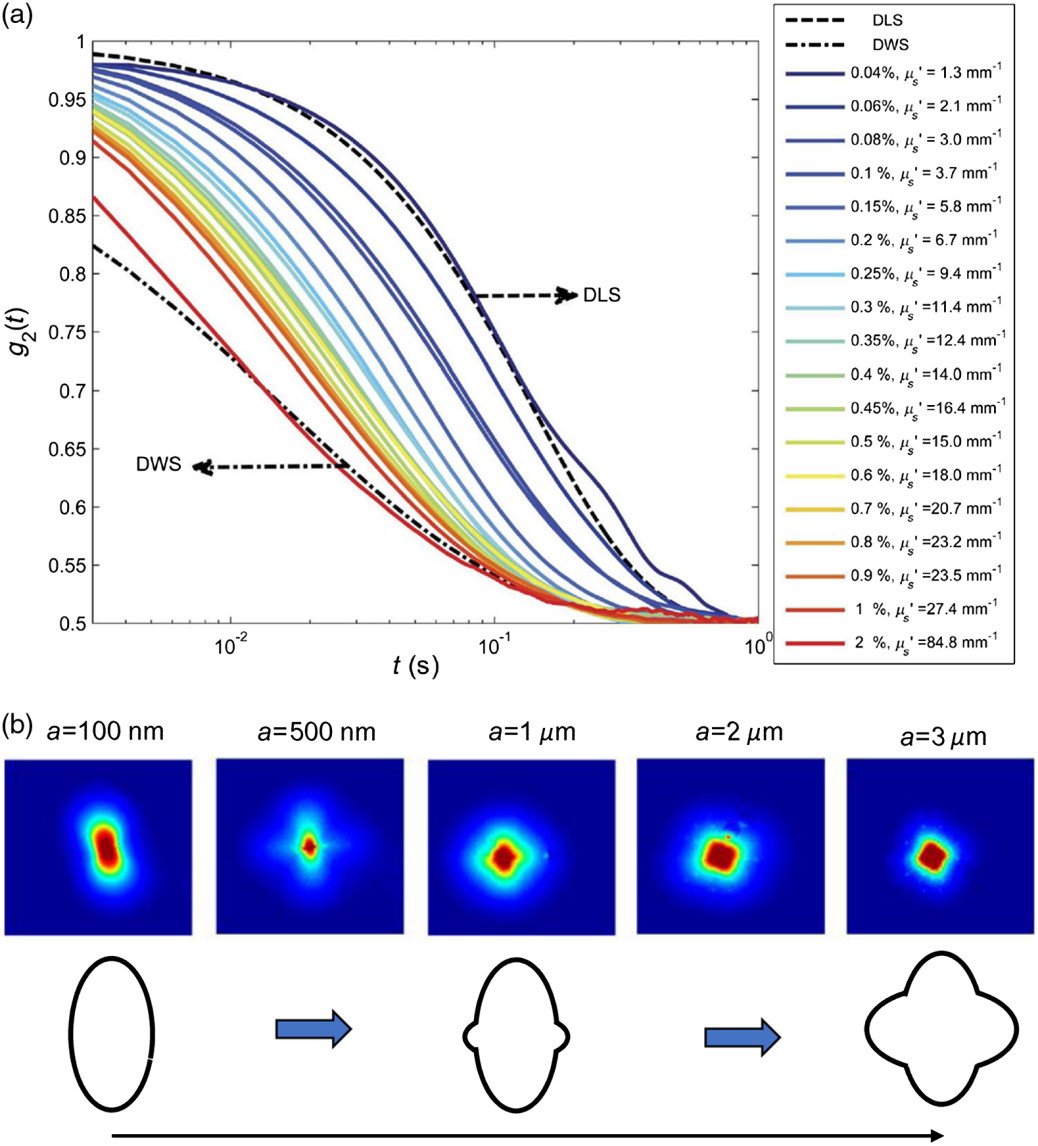
(a) Influence of optical scattering variations on speckle fluctuations. Speckle intensity temporal autocorrelation curves, $g_{2}(t)$, for aqueous glycerol mixtures of 90% glycerol, 10% water, and various concentrations of ${\mathrm{TiO}}_{2}$ scattering particles (0.04% to 2%, corresponding to ${\mu_{s}}^{\prime }$: 1.3 to 84.81/mm, N=18), along with theoretical DLS and DWS curves (dotted lines). By changing the scattering concentration, $g_{2}(t)$ curves sweep the transition area between the two theoretical limits. This data demonstrate the dependence of $g_{2}(t)$ on optical scattering in samples with identical mechanical properties (adapted from Hajjarian and Nadkarni^6^). (b) Influence of scattering particle size. Experimentally evaluated parallel-polarized DRP, remitted from mono-dispersed polystyrene bead solutions, with bead sizes ranging from 100 nm to 3  μm is shown along with the schematics of DRP shape (reproduced from Hajjarian and Nadkarni^34^).

In biological tissues, evaluating the $G^{*}$ from the measured $g_{2}(t)$ curve is not straightforward due to contributions of two main factors.^6^^,^^33^ The first factor is the influence of optical absorption and scattering, as speckle fluctuations are driven by both particles’ displacements and the number of particles encountered in photons’ paths. For example, Fig. 2(a) displays the $g_{2}(t)$ curves, obtained using Eq. (1), for aqueous glycerol mixtures of 90% glycerol and 10% water, dispersed with varying volume fractions of ${\mathrm{TiO}}_{2}$ scattering particles, ranging from 0.04% to 2%, with corresponding ${\mu_{s}}^{\prime }$ values of 1.3 to $84.8\;{\mathrm{mm}}^{-1}$ (N=18). The theoretical DLS and DWS curves (dotted lines) are also displayed. As the concentration of scattering particles is increased, the $g_{2}(t)$ curves vary between the DLS and DWS limits, for single- and rich multiple-scattering, demonstrtaing that the $g_{2}(t)$ curve depends on both mechancial and optical properties of the sample. The latter is determined by the optical absorption and reduced scattering coefficients ($\mu_{a}$, ${\mu_{s}}^{\prime }$) of the tissue.^6^^,^^33^ In other words, when ${\mu_{s}}^{\prime }$ increases, light rays experience larger number of scattering events. As such, the speckle fluctuations increase and the $g_{2}(t)$ decays more rapidly. Conversely, when $\mu_{a}$ increases, the longer optical paths that involve larger number of particles interaction are pruned due to absorption. As such, the speckle fluctuations are reduced and the $g_{2}(t)$ decays slowly. For biological tissues, $\mu_{a}$ and ${\mu_{s}}^{\prime }$ are not known beforehand. To address this, speckle frames may be temporally averaged to calculate the diffuse reflectance profile (DRP) of the specimen. From the DRP, the optical properties may be deduced in two ways. The first approach involves fitting a model, derived from light diffusion approximation, to the radial variations of DRP from the beam focus point, which yields both of $\mu_{a}$ and ${\mu_{s}}^{\prime }$.^6^^,^^33^^,^^40^ Nevertheless, $g_{2}(t)$ curve is in effect modulated by the ratio of $\mu_{a}/{\mu_{s}}^{\prime }$. This ratio maybe conveniently extracted from the total reflectance of the specimen, which can be obtained by comparing the DRP of the specimen to that of a standard reflector.^34^^,^^40^

The second factor in calculating the $G^{*}$ from MSD via the GSER is that size of scattering particles is needed.^34^ Conceptually, in addition to optical properties, size of scattering particles also modifies the speckle fluctuations, with larger particles presenting slower dynamics.^34^ Similar to optical properties, the average size of scattering particles may also be derived from temporally averaged speckle frames, i.e., diffuse reflectance. However, in this case, the speckle patterns may be acquired by placing a linear polarizing filter in front of the camera that is oriented parallel to the polarizer in the path of the illumination laser beam. Under this condition, the DRP exhibits polar angle variations that reveal the average scattering size, a.^34^^,^^35^ More specifically, when the average particle size increases in the 0.1- to 3-μm range, DRP evolves from a bi-lobular shape to a clover-like pattern, with the size of the additional lobes increasing with the particle size, as seen in Fig. 2(b).^34^ The size-dependent changes of DRP patterns are likely due to transition between isotropic Rayleigh scattering to forwardly directed Mie scattering, as the particle size increases compared to the laser source wavelength.

**Fig. 3:**
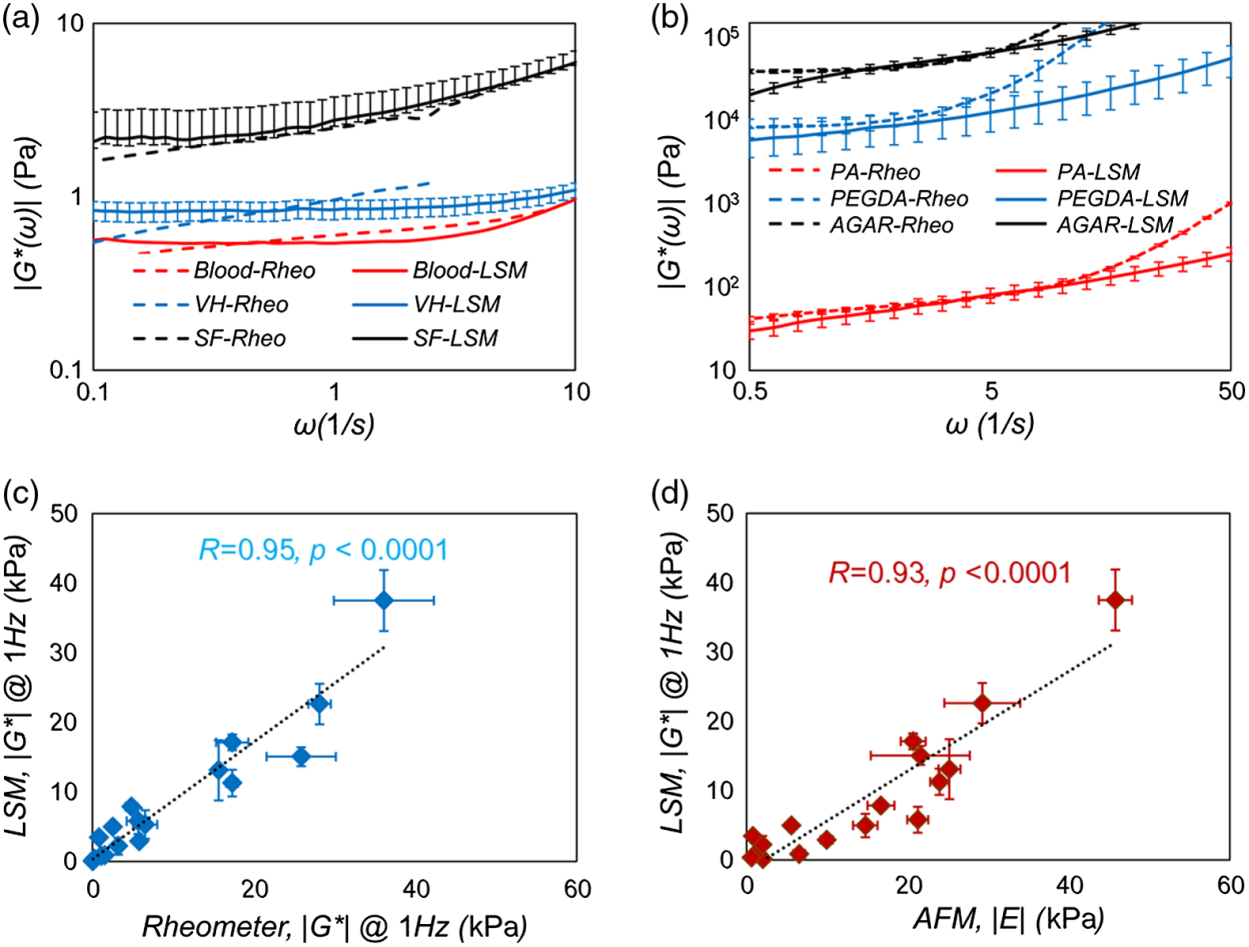
(a) $\left|G^{*}(\omega )\right|$ versus frequency obtained from LSM (solid lines) and mechanical rheometry (dashed lines) for synovial fluid, vitreous humor, and blood. Close correspondence is observed between the two measurements over the frequency range of 0.1 to 2 Hz (adapted with modifications from Hajjarian and Nadkarni^6^^,^^34^). (b) $\left|G^{*}(\omega )\right|$ versus frequency obtained from LSM (solid lines) and mechanical rheometry (dashed lines) for agarose 3%, polyacrylamide (PA, A3%, B1%), and PEGDA 10%. Close correspondence is observed between the two measurements over the frequency range of 1 to 10 Hz. Deviations at higher frequencies are due to emergence of inertial effects in conventional rheometry, which makes the results unreliable. Divergences at frequencies below 1 Hz are caused by breakdown of GSER at low strain rates together with hardware limitations of LSM, such as laser source drift, low frequency environmental vibrations, or particle size-to-pore size ratio. (c) Scatter diagram of $\left|G^{*}(\omega )\right|$ evaluated at 1 Hz obtained from LSM and conventional rheology for hydrogels (N=18). A strong, statistically significant correlation is observed between the two measurements over the moduli range of 47 m Pa to 36 kPa (r=0.95, $p<10^{-9}$). T-test analysis declared that the difference between LSM and rheometry measurements is insignificant (p=0.076). (d) Scatter diagram of $\left|G^{*}(\omega )\right|$ values at 1 Hz obtained from LSM and the indentation modulus, E, measured by AFM at the indentation rate of 2  μm/s for viscoelastic gels (N=17). Linear regression analysis declares a strong, statistically significant correlation (r=0.92, $p<10^{-7}$) for E 624 Pa to 46 kPa (reproduced with modifications from Hajjarian et al.^35^).

Figures 3(a) and 3(b) display representative examples of LSM measurements in viscoelastic biofluids and predominantly elastic hydrogels, calculated by following the steps outlined in the flowchart of Fig. 1(b) and through compensating for variations of optical properties and scattering particle size, as discussed above.^6^^,^^34^^,^^35^ The corresponding conventional mechanical rheometer measurements are also displayed, yet are only valid at lower frequencies, where the inertial effects are negligible. The onset of inertial effects is variable among different specimens and is usually lower in low viscosity liquid, as elaborated in the next section. These figures demonstrate the capability of LSM in evaluating specimens of assorted viscoelastic and elastoviscous behaviors over an expanded frequency range. Figures 3(c) and 3(d) display the strong and statistically significant correlation between LSM measurements versus rheometer and AFM in assorted hydrogel phantoms, which highlights the large dynamic range that exceeds $G^{*}:0$ to 40 kPa at $\omega =1\;s^{-1}(s^{-1}=\mathrm{Hz})$.^35^

## 4 Perspectives for Micro-Mechanical Measurements

### 4.1 Dynamic Range of Viscoelastic Modulus Measurements

Because in LSM light is multiply scattered from the tissue particles, even minute motions (fraction of a wavelength, in the order of nm) of particles, encountered within each lightpath, give rise to cumulative phase shifts that induce perceptible speckle intensity fluctuations. The rapidly fluctuating speckle patterns are captured via a high-speed camera, for a few seconds at a time to estimate the MSD. The acute susceptibility of speckle intensity to particle displacements as small as a few angstroms in LSM makes this technique capable of characterizing the mechanical properties in stiffer samples, in which particle displacements are reduced to subtle vibrations. In particular, the upper limit of viscoelastic modulus accessible to LSM is set by the size of scattering particles, a, the ability to resolve their infinitesimal motions, $\delta_{r}$, and the thermal energy, $K_{B}T$, to $G=K_{B}T/(\delta_{r}^{2}a)$.^10^ The highly sensitive multi-speckle detection scheme of LSM enables resolving displacements of δr∼Å and permits probing the viscoelastic modulus over 5 to 6 decades of magnitude, with an upper limit in the order of ∼10 to 100 kPa. In comparison, other passive techniques such as PTM resolve only considerably larger displacements via video microscopy and exhibit much smaller accessible range of $G^{\prime }$ in the order of a few Pa.^24^^,^^26^ These distinct features of LSM permit extending the passive micro-rheology to the context of stiffer biological tissues, in which intrinsic scattering particles exhibit arbitrary concentrations and size distributions.

### 4.2 Frequency Limits

As observed in Figs. 3(a) and 3(b), the upper frequency limit is reached with the advent of inertial effects in the conventional rheolometry measurements. For the rheometer, the shear strain waves applied to the sample via a parallel plate decay exponentially as they penetrate the specimen because of inertial effects. The penetration depth of shear strain waves is given by $d={\left(G^{*}/(\rho {\omega_{u}}^{2})\right)}^{0.5}$, where ρ is the density and $\omega_{u}$ is the upper frequency limit, suggesting that d is inversely proportionate to the frequency. When d < typical gap size or sample thickness, the inertial effects dominate, and the measurements become unreliable. For low viscosity fluids, this correspond to upper frequency limit of $\omega_{u}<10$ and 100 Hz, as evidenced by the curves of Fig. 3.^10^^,^^41^ Likewise, in LSM the shear waves propagated by the motion of the Brownian scattering particles decay exponentially from the surface of the bead through the sample microenvironment. Nevertheless, the inertial effects only dominate when the d∼a, where a is the scattering particle size. Therefore, in LSM the small, sub-micron size of tissue scattering particles postpones the onset of inertial effect to $\omega_{u}>\mathrm{MHz}$-regime, opening a substantially larger window of timescales and frequencies, compared to both the rheometer and other micro-mechanical testing techniques.^10^^,^^41^ The upper frequency range of competing micro-mechanical measurement techniques, namely PTM, MTC, and OTAM, are 10 Hz, 16, and 15 kHz, respectively.^12^^–^^14^^,^^24^^,^^25^ While LSM may in principle probe such a wide range of sample dynamics, in practice the high-frequency limit is further influenced by the CMOS camera; when operated at high frame rate, the camera can provide finer temporal resolution extending the frequency range of LSM. In the typical studies, a frame rate of 750 fps for instance would limit the higher frequency boundary to within 100s of Hz. By employing acquisition speeds, for instance in the order of few 100 kHz, higher frequencies in the order of $10^{5}\;\mathrm{Hz}$ may be achieved, which are not accessible to standard mechanical rheometry.

The low-frequency bound, $\omega_{\ell }$, is determined by the time scale at which longitudinal modes become significant compared to the shear modes, excited in the system.^24^^,^^26^^,^^42^ In mechanical rheology, the top plate only applies a shear strain to the sample. Therefore, in principle, no lower frequency limit exists in the case of mechanical rheology. Nevertheless, in practice evaluating the $G^{*}(\omega )$ at frequencies below 0.1 Hz may require tediously long times. In LSM, on the other hand, scattering particles respond to all the thermally excited modes, including the longitudinal modes of the elastic network. At high frequencies, the viscoelastic material may be modeled as an elastic network that is viscously coupled to the surrounding liquid. Due to the incompressible nature of the viscous liquid, no compressional strain is sustained in the microenvironment and the Brownian displacements are entirely due to excited shear modes. At low strain rates, the motion of network and liquid is decoupled and the liquid drains freely through the network. The onset of longitudinal modes is set by the ratio of sample elasticity and viscosity, as well as the ratio of network mesh to Brownian particle size, as $\omega_{\ell }∼G^{\prime }\xi^{2}/\eta a^{2}$. Here, $G^{\prime }$ is the elastic modulus of the material, ξ is the pore size, η is the viscosity of the specimen, and a is the scattering particle size. This translates into a frequency of about 0.159 Hz for a typical soft material where elastic modulus is about 100 times larger than the viscosity, and the mesh size is one-tenth of the radius of the embedded probe.

### 4.3 Viscous and Elastic Moduli and their Frequency Dependence

The LSM evaluates the complex $G^{*}(\omega )$ and is capable of measuring both the elastic (storage) and viscous (loss) moduli as a function of frequency that form the real and imaginary parts of the complex modulus, i.e., $G^{*}(\omega )=G^{\prime }(\omega )+iG^{\prime \prime }(\omega )$. More specifically, once the $G^{*}$ is evaluated through the GSER equation, these two moduli may be expressed as

$$G^{\prime }(\omega )=|G^{*}(\omega )|\times \cos(\frac{\pi }{2}\alpha (\omega ))\;G^{\prime \prime }(\omega )=|G^{*}(\omega )|\times \sin(\frac{\pi }{2}\alpha (\omega )),$$ (4)

where α is the log–log slope of the MSD at ω=1/t. By affording the ability to measure both viscous and elastic moduli, the LSM surmounts the other micro-mechanical elastography techniques, such as QME, that only evaluate the elastic response of the tissue microenvironment. This is particularly important in the context of cellular mechanobiology studies, where the interplay between micro-scale elastic and viscous moduli of cellular microenvironment is hypothesized to modulate the oncogenic signaling pathways in terms that may not be predicted solely based on the elastic properties.^43^^,^^44^ Moreover, in the context of whole tissue, the complex modulus $G^{*}$ may not always be explained by $G^{\prime }$ alone, and the contribution of loss and storage moduli to the perceived “stiffness” depends on the specific tissue type and the time-scale/loading frequency of measurements. The LSM in principle permits evaluating the various aspects of viscoelasticity and frequency-dependence, including the power-law behavior of $G^{\prime }$ and $G^{\prime \prime }$, $G^{*}$ (i.e., $G∼\omega^{\alpha }$), and the frequency dependence of loss tangent ($G^{\prime \prime }/G^{\prime }=\tan(\delta =\pi /2\alpha ))$, which present invaluable diagnostic yields. For instance, the ability to measure both elastic and viscous moduli, together with the large frequency range of LSM, permits identifying the cross-over frequency, $\omega_{c}$, at which the mechanical properties transition from elastic to viscous (sol–gel transition). The onset of $\omega_{c}$, i.e., transition to viscous behavior, is expected to be smaller in cells compared to the ECM.^14^ In addition, a reduced $\omega_{c}$ is hypothesized to be a critical indicator of malignant behavior.^14^^,^^45^

When using the algebraic approximation of GSER in LSM, we inherently assumed that MSD behavior in time domain and $G^{*}$ frequency-dependence is in symmetry. Therefore, the log-slope of MSD, i.e., α, directly returns both the frequency-dependent exponent of $G^{*}$ power-law behavior and the loss tangent of the $G^{*}(\omega )$, as δ=π/2α. Therefore, the power-law exponent may be readily probed from the MSD and no additional curve-fitting steps are needed. For a purely elastic material, particle displacements reduce to subtle vibration, MSD is constant, and α=0, which implies that δ=0. On the other hand, for a purely viscous liquid, particle displacements are diffusive, MSD grows linearly with time, α=1, and δ=π/2. Therefore, when MSD is only due to passive Brownian motion, a physically realistic range for α is 0 to 1. Nevertheless, bulk motion of tissue due to environmental vibrations could induce actively driven, super-diffusive displacements and increase α beyond 1.^46^ For instance, in the extreme case of a laminarly flowing purely viscous biofluid α reaches 2. Therefore, even minute drifts that are not perceptible in magnitude of MSD, and in turn, $G^{*}$ could significantly influence the α and in turn the evaluated $G^{\prime }$ and $G^{\prime \prime }$. Thus, an effective vibration cancellation mechanism is imperative for accurately measuring $G^{\prime }$ and $G^{\prime \prime }$ in LSM.

## 5 LSM in Cancer Mechanobiology Research

The stiffness of the extracellular matrix or tumor cell substrate has been frequently used to explain the mechano-regulation of oncogenic signaling pathways that drive the hallmarks of cancer, such as proliferation, morphogenesis, migration, and invasion, as well as vascular modulation and immune response alterations^47^ Cancer mechanobiology research has thus utilized mechanical testing methods such as conventional rheometry that measures the bulk or average stiffness of the ECM. The tumor microenvironment, however, is far from a homogeneous solid.^2^^,^^48^ It is speculated that cellular mechano-sensors perceive both the elastic stiffness and viscous dissipation at the microscale, and respond to the superposition of these cues, by either activating or suppressing their malignant traits, in terms that are not sufficiently explained by the baseline bulk stiffness.^43^^,^^44^ Micromechanical heterogeneity of the tumor microenvironment limits the uniform and effective penetration of the cytotoxic drugs around and within the tumor and thus affects the treatment outcome.^49^^–^^51^ It further restricts the even distribution of oxygen and nutrient within the tumor, and thus maintains a hypoxic niche for proliferation of drug-resistant cancer stem cells.^52^

**Fig. 4:**
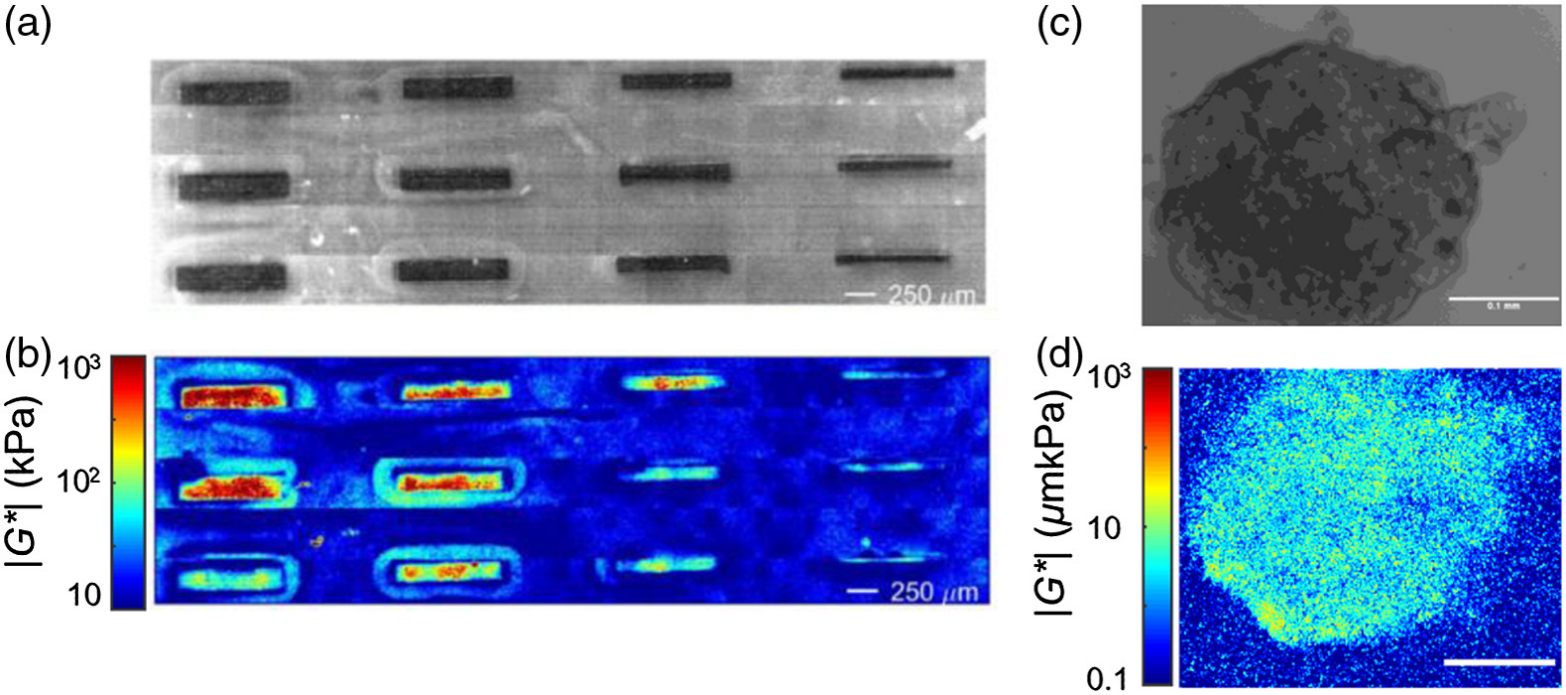
(a) Bright-field image of a micro-fabricated composite PDMS-PEGDA phantom. A total of 12 PDMS bars are visible within the PEGDA background. The bars in successive columns are 1-mm long and 200-, 150-, 100-, and 80-μm wide, respectively. (b) Spatially resolved $G^{*}$, evaluated at 100 Hz. In the color-bar, the moduli range of 10 kPa to 1 MPa is represented by blue to red hues. Significant contrast is observed between stiff PDMS bars and the PEGDA 5% background at all length-scales. Scale bars: 250  μm. (Adapted with modification from Hajjarian et al.^35^) (c) Bright-field image of a micro-metastatic ovarian cancer nodules, and (d) viscoelastic modulus, $\left|G^{*}\right|$, evaluated by LSM. Mechanically stiffer regions are apparent even at the cellular levels and a mechanical transformation from surrounding matrix is observed. These results establish that LSM is able to map the ECM mechanical properties at smaller length scales, in the order of a few cell clusters. Scale bars: 100  μm.

In this context, the capability of the LSM for high-resolution, non-contact mapping of tumor micro-environment within *in-vitro* culture systems of cancer cells grown on engineered ECM substrates, under sterile condition is instrumental for providing a renewed and refined perspective of tumor mechanobiology. Furthermore, the susceptibility of LSM measurements to sub-wavelength scattering particle displacements, and in turn sensitivity to moduli changes of less than 1 Pa, makes this technology highly desirable for longitudinal studies of micro-mechanical modulation. Figures 4(a) and 4(b) demonstrate the spatial resolution of LSM micro-mechanical maps in a micro-fabricated polydimethylsiloxane-polyethylene glycol diacrylate (PDMS-PEGDA) phantoms, featuring highly stiff PDMS bars of 1-mm long, and 200, 150, 100, 80, and 60  μm wide within a background of compliant PEGDA 5% gel.^35^ From this figure, it is evident that LSM resolves micro-mechanical features in the order of a few 10s μm. This is comparable with the length-scales of cell-ECM micro-mechanical interactions and speaks to utility of LSM for probing mechanobiological processes at the interface of cells and their microenvironment. We have previously conducted a longitudinal study of micro-mechanical remodeling in an *in-vitro* OvCar5 cells, embedded in a Matrigel ECM,^53^ as they aggressively migrated and assembled into large multicellular 3D tumor micro-spheroid clusters in the span of 21 days (unpublished results). Figures 4(c) and 4(d) displays the bright field image of a micro-metastatic ovarian cancer nodules at the seventh day of the study, along with the corresponding spatial map of $\left|G^{*}\right|$, evaluated by LSM. Mechanically stiffer regions are apparent even at the cellular levels and a mechanical transformation from surrounding matrix is observed. An important consideration when conducting LSM in cell culture systems is the influence of active intra-cellular motion on the speckle intensity fluctuations. Prior studies by other groups have investigated the distinctions between the time scales of Brownian motions and these active processes. In particular, speckle fluctuations caused by diffusion of gold particles in the ECM and intracellular adenosine triphosphate (ATP) motors have been previously studied.^54^ Accordingly, the time scale of ATP-driven speckle decorrelation is in the order of 5 s and is captured only when the frame rate is <1  Hz. Given that LSM acquires speckle images at much higher frames rates in the order of 100s and 1000s of frames per second, the contribution of non-Brownian dynamics to the LSM measurements is minimal. We independently verified this by evaluating 3 plane 4% agarose gels and 3 others seeded with 3T3 fibroblast cells. T-test declared no significant difference in $\left|G^{*}(\omega )\right|$, evaluated at ω=1 and 100 Hz between the two groups (p=0.23). These studies open exciting opportunities for future applications of LSM to obtain insights on the mechanically mediated cross-talk between cells and their 3D microenvironment with high resolution and sensitivity.

LSM may further assist with the development of new lines of therapeutics that focus on alleviating micro-mechanical abnormalities. The traditional understanding of tumor mechanobiology has suggested several mechano-therapies that aimed to regress the course of cancer by reducing the tumor stiffness. For instance, anti-fibrotic reagents and matrix metalloproteinase inhibitors are proposed to target the ECM micromechanical properties.^55^^,^^56^ Nevertheless, these approaches largely failed in clinical trials, likely due to extensive toxicity associated with off-target interruption of tissue functions in healthy organs.^2^^,^^57^ LSM affords the capability to evaluate the micro-mechanical properties of tumor cells cultured on engineered substrates, and permits investigating the interaction and interplay of cell–ECM viscoelastic properties. In this way, it could assist developing successful therapeutic strategies based on both restoration of the ECM micro-mechanical properties and disruption of the aberrant cellular responses to ideally strike all the modifiable targets at the same time.^2^ By quantifying the changes in micro-mechanical properties of the ECM, LSM could facilitate the development of such optimal interventions for clinical benefit across multiple diseases.

## 6 Summary and Conclusion

We recapitulated the ability of LSM for evaluating key quantitative metrics of elastic, viscous, and viscoelastic moduli as well as loss tangent of tissues and biomaterials. We further renumerated the unique features of this technology, including large dynamic-range, unparalleled frequency limits, and exquisite sensitivity, which permit investigating fundamental questions on micromechanical aspects of cancer etiology and drug resistance. Beyond cancer research applications, the LSM can also be used to study mechanosensitive processes pertinent in wound healing, atherosclerosis, as well as neurodegenerative and orthopedic diseases.^49^^,^^58^^–^^61^ We envision these insights will open new perspectives for rheology-informed disease prognosis and drug development for improved therapeutic efficacy in the future.
